# Abnormalities in microbial composition and function in infants with necrotizing enterocolitis: A single-center observational study

**DOI:** 10.3389/fped.2022.963345

**Published:** 2022-10-21

**Authors:** Huan Huang, Qian Peng, Yuli Zhang, Ying Li, Nanqu Huang, Miao Duan, Bo Huang

**Affiliations:** ^1^ Department of Pediatrics, Third Affiliated Hospital of Zunyi Medical University (The First People’s Hospital of Zunyi), Zunyi, China; ^2^ National Drug Clinical Trial Institution, Third Affiliated Hospital of Zunyi Medical University (The First People’s Hospital of Zunyi), Zunyi, China

**Keywords:** necrotizing enterocolitis, 16S rRNA, intestinal microbiota, neonatal, PICRUSt2

## Abstract

**Objective:**

To investigate the features and functions of the intestinal microbiota in neonates with necrotizing enterocolitis (NEC) in a single center in China.

**Methods:**

We collected clinical information and stool samples from 19 participants in our center, including 9 infants with necrotizing enterocolitis and 10 control infants. DNA was extracted from the samples, and 16S rRNA gene sequencing was used to analyse the participants' gut microbiota. Functional prediction was achieved using PICRUSt2.

**Results:**

Alpha diversity analysis found that similar levels of bacterial richness and diversity were found in the gut microbiota of infants with NEC and control infants (*P *= 0.1800), whereas beta diversity analysis suggested that the overall structures of the gut microbiota were significantly different (*P *= 0.0020). The *Mann–Whitney U test* of bacterial composition and abundance analysis revealed that the abundance levels of *Proteobacteria* (*P *= 0.03049) and *Firmicutes* (*P *= 0.01011) significantly differed between the two groups at the phylum level. *Proteobacteria* was the most abundant phylum in the NEC group. At the genus level, the abundance levels of *Enterococcus* (*P *= 0.0003), *Streptococcaceae* (*P *= 0.0109) and *Lactobacillales* (*P *= 0.0171) were significantly decreased in infants with NEC. Furthermore, the linear discriminant analysis effect size (LEfSe) method showed 12 bacterial taxa with significant differences in relative abundances in the two groups. Interestingly, members of *Proteobacteria* were enriched in NEC samples. In addition, functional prediction suggested that the microbial changes observed in infants with NEC resulted in a decline in galactose metabolism, the pentose phosphate pathway, fructose and mannose metabolism, amino sugar and nucleotide sugar metabolism, glycolysis/gluconeogenesis, starch and sucrose metabolism, and phosphotransferase system (PTS) pathways (*P *< 0.05).

**Conclusions:**

Our study shows the compositional and functional alterations of the intestinal microbiota in NEC, which will help demonstrate the relationship between the gut microbiota and NEC pathogenesis.

## Introduction

Necrotizing enterocolitis (NEC) is one of the most common gastrointestinal emergencies in neonatal intensive care units (1). The main clinical manifestations are abdominal distension, vomiting, diarrhoea, and blood in the stool (2). Despite many efforts by researchers, current clinical predictors of NEC remain unclear and lack sufficient specificity, and we need better diagnostic tools to diagnose NEC early and improve its associated morbidity. NEC is considered a disease with multifactorial aetiology, and preterm birth, feeding patterns, gut dysbiosis, and antibiotic use play important roles in the development of NEC (3–6). Despite accumulating evidence that gut microbiota dysregulation plays an important role in the pathogenesis of NEC, the underlying mechanisms remain unclear.

With the application and development of high-throughput sequencing technology, we have obtained a deeper understanding of the relationship between NEC and the gut microbiome. Current research not only focuses on the impact of individual microbes on the disease but also looks at the microbial community as a whole. Due to individual differences and diverse sequencing platforms, no specific species or uniform microbial signatures have been demonstrated to be causally related to NEC.

Neonates, especially premature infants, have intestinal local immune systems that are not yet fully matured and thus are susceptible to the influence of intestinal flora; intestinal flora imbalance may be an important factor in the pathogenesis of NEC (7, 8). Most studies reporting on the pathogenesis of NEC have primarily studied extremely preterm infants. However, in developing countries, compared to developed countries, only 10% of infants with NEC born before 28 weeks survive, and most infants with NEC are born beyond 28 weeks (9). In addition, existing related studies mainly focused on 16S rRNA gene sequencing, but further studies on microbial functions are still lacking.

Given the possible role of the gut microbiota in infants with NEC, we conducted a prospective observational study of 19 individuals, including 11 control infants and 9 infants with NEC, and we used 16S rRNA sequencing and PICRUSt2 functional prediction to investigate the role of gut microbial composition and function in infants with NEC.

## Materials and methods

### Ethics statement

This study was conducted from December 2018 to October 2020 and was approved by the Ethics Committee of The Third Affiliated Hospital of Zunyi Medical University (The First People’s Hospital of Zunyi) (Ethics No. 2018-058). All parents of participants were informed of the nature of the study before sample collection and were asked to provide written informed consent.

### Inclusion criteria

1. Diagnosis of necrotizing enterocolitis (NEC) made by a neonatologist based on the modified Bell staging criteria (10).
2. Control neonates who matched the infants with NEC in gestational age, sex, birth method, etc.
3. Approval obtained from participants’ parents or legal representative.

### Exclusion criteria

1. Infants with known congenital malformations of the digestive tract, such as gastroschisis and intestinal atresia.
2. Spontaneous intestinal perforation, which was confirmed by a combined radiological and surgical diagnosis.
3. Use of probiotics before the first stool specimen collection.
4. Incomplete clinical data or failure to follow the procedures to store samples.

### Collection of samples

All faecal samples were collected within 48 h of infant admission to the NICU. Weekly stool samples were obtained from all infants who met the inclusion criteria until they were discharged from the hospital. When infants presented with NEC symptoms and were diagnosed with NEC (Bell stage II and III), they were considered the NEC group, and their stool samples were collected within 48 h and again 1–2 weeks after treatment. To exclude confounding factors, control infants who matched the infants with NEC in gestational age, sex, birth method, etc. All infants who died or for whom treatment was stopped were withdrawn the study. Samples were stored at −20°C within 24 h of collection and then transferred to the laboratory and stored at −80°C until they were sent to Shanghai Majorbio Bio-pharm Technology Co., Ltd. for 16S rRNA analysis.

### DNA extraction, PCR ampliﬁcation, and illumina MiSeq sequencing

According to the instructions of the QIAamp Stool Mini Kit (QIAGEN, Germany), total bacterial genomic DNA in stool samples was extracted, and PCR ampliﬁcation was performed on the V3–V4 region of the bacterial 16S rRNA gene. The sequence of the forwarding primers used was 338F (5′ACTCCTACGGGAGGCAGCA-3′), and the reverse primer used sequence was 806R (5′-GGACTACHVGGGTWTCTAAT-3′). The Illumina MiSeq Sequencing PE300 platform was selected for sequencing analysis, and the raw sequencing data were saved in FastQ format (the raw data have been uploaded to NCBI, PRJNA843530).

### Bioinformatic processing

The data were analysed on the Majorbio Cloud Platform (www.majorbio.com). Alpha diversity analysis was used to show the richness and diversity of the community. Beta diversity analysis was performed mainly by using principal coordinate analysis (PCoA) to show the differences in microbial community composition of each sample and combined with ANOSIM to reflect the community differences between groups and within each group. A Circos circle diagram (Circos-0.67–7 software, http://circos.ca/) was used to visualize the composition ratio of species in each group of samples. Linear discriminant analysis effect size (LEfSe) analysis was used to detect differences in species abundance between groups, and LDA was combined to estimate the impact of these different species on group discrimination. Using the PICRUSt2 method, the Kyoto Encyclopedia of Genes and Genomes (http://www.genome.jp/kegg/) was used to compare the different metabolic pathways between groups and to predict the function of the microbial community.

### Statistical analysis

SPSS 22.0 software was used to analyse the clinical data of the participants. Quantitative variables with a nonnormal distribution are presented as the median/interquartile range and were compared with the *Mann–Whitney test*. Qualitative variables are presented as frequencies/percentiles and were compared with *Fisher’s exact test*. A *P*–value <0.05 indicated that a difference was statistically significant.

## Results

### Participant characteristics

**Figure 1:**
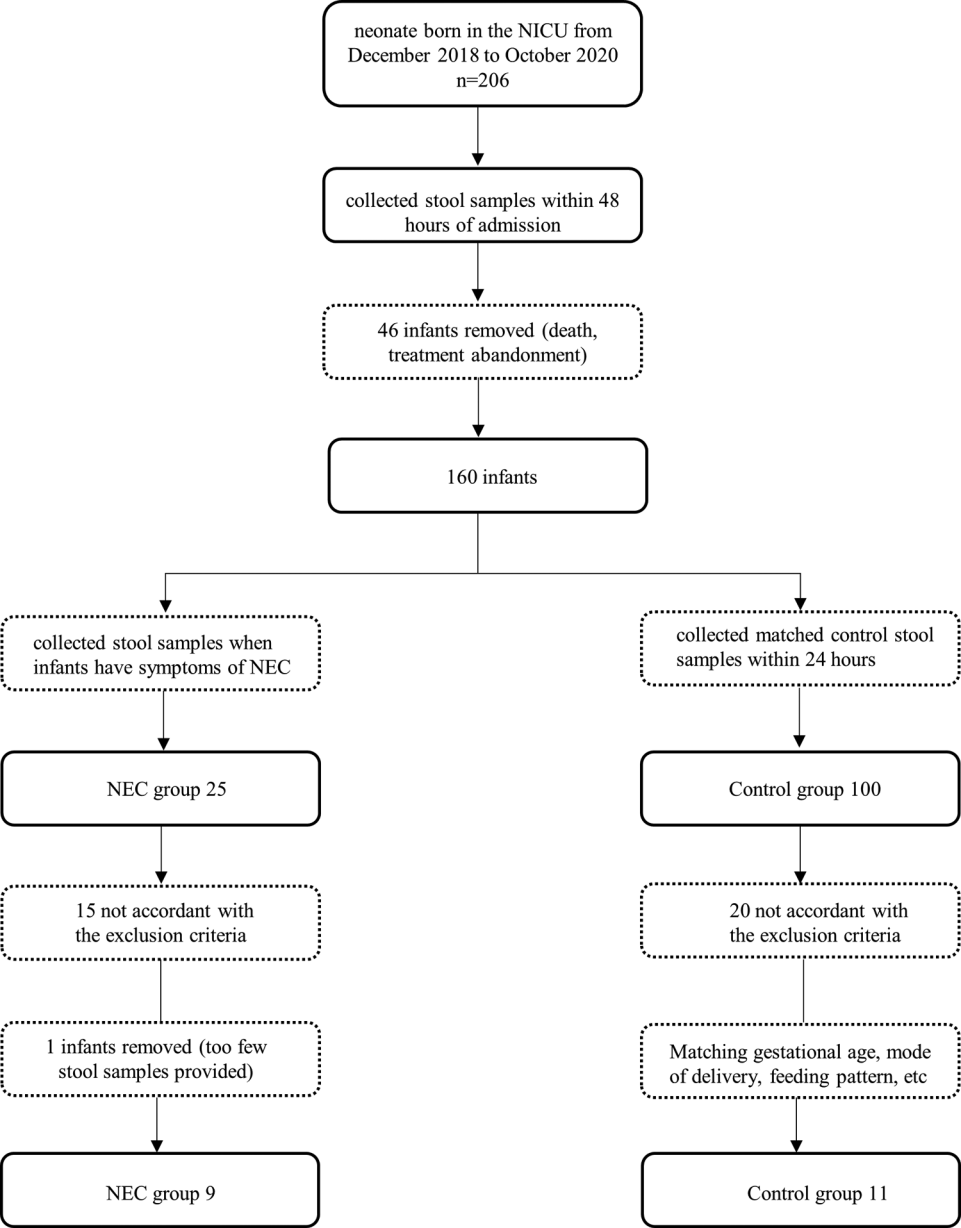
Flowchart for screening samples.

At the onset of NEC symptoms, seven samples from infants were collected. Two samples of infants were taken after NEC treatment. The control group faecal samples were collected within 48 h, which was close to the time of diagnosis in the NEC group. The specific screening process is shown in the flowchart (Figure 1). All infants with NEC were treated with antibiotics according to the severity of the disease, and 2 infants underwent surgery. Although breastfeeding is a priority, formula feeding had to be used for the control of confounding factors. For some infants with feeding intolerance, probiotics, commonly *Bifidobacterium*, are used in our center. The participant characteristics of the two groups are shown in Table 1.

**Table 1:** Participant characteristics.

Variables, description	NEC group (*n* = 9)	Control group (*n* = 10)	*P*-value (Fisher's Exact Test)
Birth weight (kg), median, IQ	1,750 (1,575–2,875)	2,800 (1,363–3,350)	0.01*
Gestational age (weeks), median, IQ	31.6 (28.35–37.45)	37.75 (32.03–39.05)	Ns
Sex (males/females), number (%)	4 (44.4%)/5 (55.6%)	6 (60%)/4 (40%)	ns
Caesarean section, number (%)	6 (66.7%)	7 (70%)	ns
Apgar Score (1 min), median, IQ	9 (7.5–9.5)	9 (9–10)	ns
Antibiotics, number (%)	9 (100%)	6 (60%)	ns
Probiotics, number (%)	5 (55.6%)	3 (30%)	ns
NEC treatment (surgical), number (%)	2 (22.2%)	–	ns
Mortality associated with NEC	–	–	–

ns, not signiﬁcant (*P *> 0.05); NEC, necrotizing enterocolitis; IQ, interquartile range.

*means significant (*P*<0.05).

### Comparison of alpha diversity and beta diversity in the NEC and control groups

**Figure 2:**
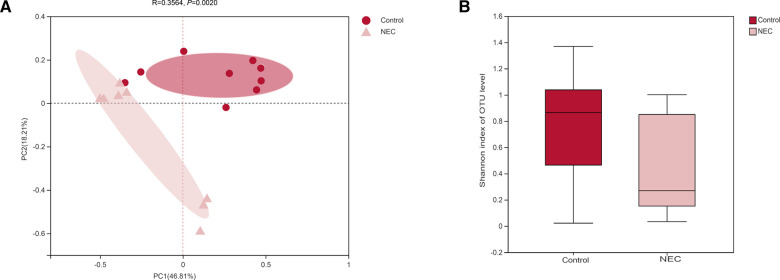
(**A**) principal component analysis (PCoA) of the gut microbiota; (**B**) shannon diversity index of the gut microbiota, *Mann‒Whitney U test*.

To study the differences in the gut microbiota between NEC patients and controls, we performed beta diversity analysis to assess the overall differences and similarities in microbial population structure. PCoA demonstrated that the microbial composition in the two groups was significantly different based on Bray‒Curtis distances at the OTU level (*R* = 0.3564, *P *= 0.0020, Figure 2A). In addition, as measured using alpha diversity, we found that the Shannon index was not significantly different between the NEC and control groups; however, a declining trend was observed in the NEC group (*P *= 0.1800, Figure 2B). In summary, the above results showed that compared with the control group, similar levels of bacterial richness and diversity were found in the gut microbiota of infants with NEC, whereas the overall structures of the gut microbiota of infants with NEC and control infants were significantly different, indicating that infants with NEC show gut microecology dysbiosis.

### Different bacterial composition and abundance in the NEC and control groups

**Figure 3:**
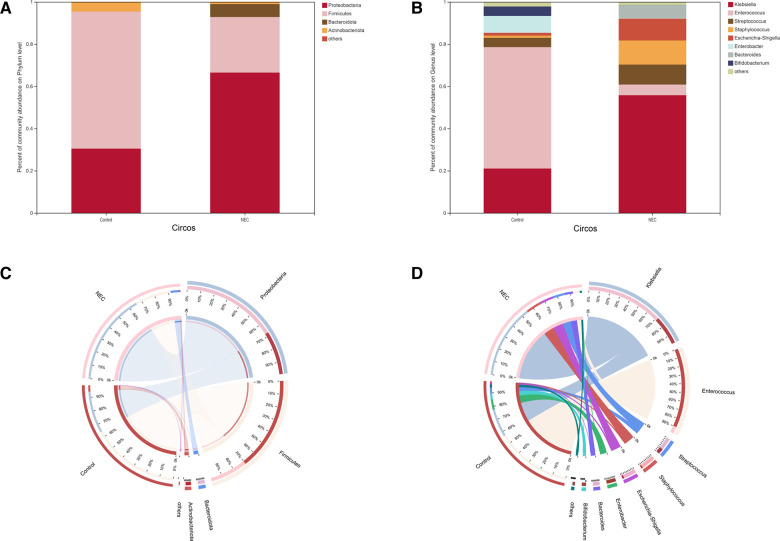
Community composition analysis showing the species composition of each group of samples: (**A**) phylum level, (**B**) genus level; composition and distribution of dominant species in the sample: (**C**) circos diagram of phylum level, (**D**) circos diagram of genus level. The abundance levels of certain bacteria are associated with NEC

**Figure 4:**
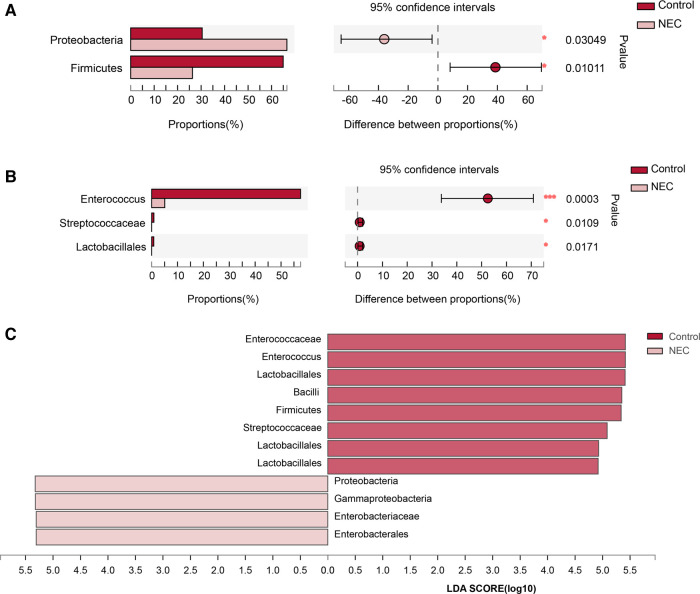
Analysis of significant species differences: (**A**) Species with significant differences at the phylum level; (**B**) Species with significant differences at the genus level; (**C**) LDA scores for gut microbiota.

To investigate the specific changes in the microbiota in samples from infants with NEC, we assessed the relative abundance of the microbial composition in all samples. At the phylum level, we observed 4 phyla in the NEC and control groups. *Proteobacteria*, *Firmicutes, Bacteroidetes, and Actinobacteria* were the most dominant phyla in each group (Figures 3A,C). Additionally, significant differences in the relative abundance of *Proteobacteria (P *= 0.03049) and *Firmicutes* (*P *= 0.01011) were observed between the two groups (Figure 4A, *Mann–Whitney test*). These findings indicate that infants with NEC have a relatively higher abundance of *Proteobacteria* in the gut.

At the genus level, a total of 8 genera were identified. In Circos analysis, *Klebsiella* was found to be the most abundant genus in the NEC group, whereas *Enterococcus* was the dominant genus in the control infants. In addition, a higher abundance of *Staphylococcus*, *Streptococcus*, *Escherichia-Shigella*, and *Bacteroides* and a lower abundance of *Enterobacter* and *Bifidobacterium* were observed in the NEC group than in the control group (Figures 3B,D). Of note, the abundance levels of *Enterococcus* (*P *= 0.0003), *Streptococcaceae* (*P *= 0.0109) and *Lactobacillales* (*P *= 0.0171) were significantly decreased in infants with NEC (Figure 4B, *Mann–Whitney test*). Some phyla/genera, whose relative abundance was lower than 0.01, were clustered into a separate group named others.

To identify the specific bacteria associated with NEC, we performed LFfSe analysis to compare the compositions of the gut microbiota of infants with NEC and control infants from the phylum to the genus level. In total, there were 12 bacterial taxa for which significant differences in relative abundances in the two groups at the phylum (*n* = 2), class (*n* = 2), order (*n* = 2), family (*n* = 3), and genus (*n* = 3) levels were observed (LDA >  2, Figure 4C). Members of *Firmicutes* were prevalent in the control infants, whereas members of *Proteobacteria* were enriched in the NEC samples. Therefore, these taxa may be used as biomarkers to discriminate NEC patients.

### Microbial functional dysbiosis in NEC

**Figure 5:**
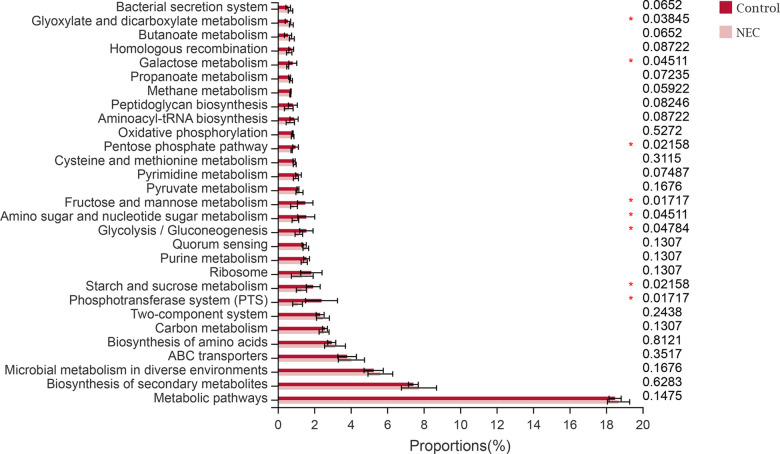
PICRUSt2 analysis of the abundance of the top 30 KEGG pathways.

To study the functional alterations of the microbial communities in NEC, we determined the functional potential of the gut microbiota from the 16S rRNA data by PICRUSt2. The analysis identiﬁed 8 PICRUSt2 KEGG (level 3) categories that signiﬁcantly differed between the two groups. Of note, we found that the majority were related to metabolism, and pathways involving glyoxylate and dicarboxylate metabolism were signiﬁcantly increased in the microbiome of infants with NEC, whereas galactose metabolism, the pentose phosphate pathway, fructose and mannose metabolism, amino sugar and nucleotide sugar metabolism, glycolysis/gluconeogenesis, and starch and sucrose metabolism pathways were signiﬁcantly decreased (*P *< 0.05). In addition, the phosphotransferase system (PTS) was significantly decreased in infants with NEC (*P *< 0.05, Figure 5).

## Discussion

Necrotizing enterocolitis is a gastrointestinal disease of unknown aetiology whose pathogenesis is currently unclear. Multiple studies have shown that necrotizing enterocolitis (NEC) mainly affects low birth weight infants, and the reported death rate is between 15% and 30% (11–15). Stoll et al (16). performed a retrospective analysis of 10,877 very low birth weight, very low gestational age neonates, and they found an incidence of NEC of 8.9% in this population. Our study found that the birth weight of infants with NEC was mainly low (*P* < 0.05), which was similar to the findings reported in these studies to a certain extent (12, 17, 18).

Many studies have confirmed that the microbiome of infants with NEC differs in number and diversity compared to that of healthy infants. However, to date, studies on the diversity or composition of the microbiome associated with the disease have not been conducted. Early studies based on culture techniques showed that compared to infants without NEC, infants with NEC had changes in faecal bacteria, including an increase in *Escherichia coli* and a decrease in *Streptococcus faecalis* and *Staphylococcus* (19). A large multicentre study found an increased abundance of *Gammaproteobacteria* prior to a diagnosis of NEC (20). In addition, Morrow et al. reported that the majority of preterm infants with NEC had lower microbial diversity than other infants of the same gestational age and an increased abundance of *Proteobacteria* and *Firmicutes* before further development of NEC (21, 22). In contrast, Norman et al. (23) did not find that the microbial diversity index decreased in their research, and the authors argued that a lack of bacterial diversity is not necessarily associated with the development of NEC. In this study, we found no differences in overall diversity between infants with NEC and control infants, but we found a decreasing trend in the overall diversity in infants with NEC, although the difference was not statistically significant.

Close interactions among the gut microbiota, epithelial cells, and immune cells are critical for maintaining gut homeostasis (24). Lu et al. (25) suggested that LPS produced by *Proteobacteria* may be recognized by TLR4 receptors in the gut, leading to the transcription of various proinflammatory and anti-inflammatory cytokines and chemokines and triggering intestinal inflammation and intestinal cell damage, resulting in the development of NEC in infants. In this study, we also found an increase in *Proteobacteria* and a decrease in *Firmicutes* in infants with NEC at the phylum level (*P* < 0.05). This suggests that the increase in *Proteobacteria* and the decrease in *Firmicutes* in faeces may serve as biomarker signals for the dysbiosis typical of NEC. In addition, we found that the abundance of *Enterococcus* (*P *< 0.0005), *Streptococcaceae* (*P *< 0.05), and *Lactobacillales* (*P *< 0.05) was significantly reduced in infants with NEC at the genus level. *Enterococcus*, a type of bacteria in the gut, is an important part of the healthy human gut microbiome, and *Enterococcus* can cause histopathological changes only under certain conditions, leading to infection (26). As mentioned by many authors, *Enterococcus* is classified as a source of infection in hospitalized patients, and these infections can sometimes be life-threatening (27). Intriguingly, several studies reported that *Enterococcus faecalis*, an important species of *Enterococcus*, was reduced in NEC (28). *Enterococcus faecalis* can inhibit the damage to intestinal villi and crypt atrophy caused by *Salmonella Enteritidis* colonization in the intestine, regulate intestinal microbial function, and improve intestinal mucosal barrier damage (29). In addition, *Enterococcus* is considered a probiotic to treat antibiotic-associated diarrhoea and irritable bowel syndrome and to improve host immunity (30).

*Lactobacillus* has been accepted as a probiotic (31–33) that can inhibit proinflammatory factors, increasing the ratio of regulatory to pathogenic T cells, leading to anti-inflammatory effects and repairing the damaged intestinal barrier (34). According to reports, probiotic treatment of very low birth weight infants increased the relative abundance of *Streptococcus* and *Lactobacillus* and decreased the relative abundance of *Enterobacteriaceae*, which improved the survival rate of these infants (35). Overall, we support the idea that a single microbial species is not the cause of NEC and that NEC may be caused by a combination of multiple species.

Through functional analysis, we found that infants with NEC exhibited an enrichment of the glyoxylate and dicarboxylate metabolism KEGG pathways, and galactose metabolism, the pentose phosphate pathway, fructose and mannose metabolism, amino sugar and nucleotide sugar metabolism, glycolysis/gluconeogenesis, starch and sucrose metabolism, and phosphotransferase system (PTS) KEGG pathways were significantly impaired (*P *< 0.05). By comparing the difference in metabolomics in serum before and after intestinal infection, Pan Lei's team found that the local innate immune response caused by intestinal infection can cause the activation of the polyol metabolic pathway (the polyol metabolic pathway is often elevated in bacterial infections, hepatitis C or diabetes), resulting in the decline of glucose and galactose metabolism levels (36). Furthermore, some studies have found that glucose-6-phosphate is produced through gluconeogenesis, glycogenolysis, and glycolysis pathways (37, 38) and further produces abundant nicotinamide adenine dinucleotide phosphate through the pentose phosphate pathway. High levels of glutathione promote inflammatory macrophages to mediate inflammatory responses. On the other hand, glycogen metabolism also increases UDP-glucose levels and P2Y14 receptors in macrophages, and the UDPG/P2Y14 signalling pathway not only upregulates STAT1 expression by activating RAR*β* but also promotes STAT1 phosphorylation by downregulating phosphatase TC45, thereby regulating inflammatory responses (39). Therefore, alterations in the function of the gut microbiota may play an important role in the pathogenesis of NEC.

### Limitations

Infants with NEC require short-term fasting, which increases the difficulty of sample collection. Therefore, our study population was small, and we included only 9 infants with NEC. Another limitation is that our study was a single-centre study, and the local geographic region may also influence the composition of the microbiota. Therefore, an independent multicentre study is needed to validate the findings of this study. Our data may provide some guidance for the therapy of NEC to alleviate the further development of NEC.

## Conclusion

Infants with NEC are susceptible to low birth weight. A single microbial species is not the cause of NEC, and NEC may be caused by a combination of multiple species. Abnormal gut microbiota composition and function may be important factors in the pathogenesis of NEC. Targeting the characteristics and functions of the gut microbiota may help us to further study the pathogenesis of NEC.

## Data Availability

The datasets presented in this study can be found in online repositories. The names of the repository/repositories and accession number(s) can be found below: https://www.ncbi.nlm.nih.gov/, PRJNA843530.

## References

[1] Patel RM Kandefer S Walsh MC Bell EF Carlo WA Laptook AR Causes and timing of death in extremely premature infants from 2000 through 2011. N Engl J Med. (2015) 372(4):331–40. 10.1056/NEJMoa1403489 25607427 PMC4349362

[2] Thänert R Keen EC Dantas G Warner BB Tarr PI. Necrotizing enterocolitis and the microbiome: current status and future directions. J Infect Dis. (2021) 223(12 Suppl 2):S257–63. 10.1093/infdis/jiaa604 33330904 PMC8206796

[3] Eaton S Rees CM Hall NJ. Current research on the epidemiology, pathogenesis, and management of necrotizing enterocolitis. Neonatology. (2017) 111(4):423–30. 10.1159/000458462 28538238

[4] Isani MA Delaplain PT Grishin A Ford HR. Evolving understanding of neonatal necrotizing enterocolitis. Curr Opin Pediatr. (2018) 30(3):417–23. 10.1097/MOP.0000000000000629 29601338

[5] Calatayud M Koren O Collado MC. Maternal microbiome and metabolic health program microbiome development and health of the offspring. Trends Endocrinol Metab. (2019) 30(10):735–44. 10.1016/j.tem.2019.07.021 31493988

[6] Rodríguez JM Murphy K Stanton C Ross RP Kober OI Juge N The composition of the gut microbiota throughout life, with an emphasis on early life. Microb Ecol Health Dis. (2015) 26:26050. 10.3402/mehd.v26.26050 25651996 PMC4315782

[7] Claud EC Walker WA. Colonization of the premature intestine. FASEB J. (2001) 15(8):1398–403. 10.1096/fj.00-0833hyp 11387237

[8] Weiss GA Hennet T. Mechanisms and consequences of intestinal dysbiosis. Cell Mol Life Sci. (2017) 74(16):2959–77. 10.1007/s00018-017-2509-x 28352996 PMC11107543

[9] Blencowe H Cousens S Oestergaard MZ Chou D Moller AB Narwal R National, regional, and worldwide estimates of preterm birth rates in the year 2010 with time trends since 1990 for selected countries: a systematic analysis and implications. Lancet. (2012) 379(9832):2162–72. 10.1016/S0140-6736(12)60820-4 22682464

[10] Walsh MC Kliegman RM. Necrotizing enterocolitis: treatment based on staging criteria. Pediatr Clin North Am. (1986) 33(1):179–201. 10.1016/S0031-3955(16)34975-6 3081865 PMC7131118

[11] Cao X Zhang L Jiang S Li M Yan C Shen C Epidemiology of necrotizing enterocolitis in preterm infants in China: a multicenter cohort study from 2015 to 2018. J Pediatr Surg. (2022) 57(3):382–6. 10.1016/j.jpedsurg.2021.05.014 34175121

[12] Fitzgibbons SC Ching Y Yu D Carpenter J Kenny M Weldon C Mortality of necrotizing enterocolitis expressed by birth weight categories. J Pediatr Surg. (2009) 44(6):1072–5; discussion 5–6. 10.1016/j.jpedsurg.2009.02.013 19524719

[13] Horbar JD Carpenter JH Badger GJ Kenny MJ Soll RF Morrow KA Mortality and neonatal morbidity among infants 501 to 1500 grams from 2000 to 2009. Pediatrics. (2012) 129(6):1019–26. 10.1542/peds.2011-3028 22614775

[14] Nesterenko TH Baliga N Swaintek S Abdelatif D Aly H Mohamed MA. The impact of a multifaceted quality improvement program on the incidence of necrotizing enterocolitis in very low birth weight infants. Pediatr Neonatol. (2022) 63(2):181–7. 10.1016/j.pedneo.2021.10.002 34933821

[15] Yee WH Soraisham AS Shah VS Aziz K Yoon W Lee SK. Incidence and timing of presentation of necrotizing enterocolitis in preterm infants. Pediatrics. (2012) 129(2):e298–304. 10.1542/peds.2011-2022 22271701

[16] Bell EF Hintz SR Hansen NI Bann CM Wyckoff MH DeMauro SB Mortality, in-hospital morbidity, care practices, and 2-year outcomes for extremely preterm infants in the US, 2013–2018. JAMA. (2022) 327(3):248–63. 10.1001/jama.2021.23580 35040888 PMC8767441

[17] Thompson AM Bizzarro MJ. Necrotizing enterocolitis in newborns: pathogenesis, prevention and management. Drugs. (2008) 68(9):1227–38. 10.2165/00003495-200868090-00004 18547133

[18] Qian T Zhang R Zhu L Shi P Yang J Yang CY Necrotizing enterocolitis in low birth weight infants in China: mortality risk factors expressed by birth weight categories. Pediatr Neonatol. (2017) 58(6):509–15. 10.1016/j.pedneo.2016.10.004 28528756

[19] Hoy C Millar MR MacKay P Godwin PG Langdale V Levene MI. Quantitative changes in faecal microflora preceding necrotising enterocolitis in premature neonates. Arch Dis Child. (1990) 65(10 Spec No):1057–9. 10.1136/adc.65.10_Spec_No.1057 2122814 PMC1590248

[20] Warner BB Deych E Zhou Y Hall-Moore C Weinstock GM Sodergren E Gut bacteria dysbiosis and necrotising enterocolitis in very low birthweight infants: a prospective case-control study. Lancet. (2016) 387(10031):1928–36. 10.1016/S0140-6736(16)00081-7 26969089 PMC5553277

[21] Morrow AL Lagomarcino AJ Schibler KR Taft DH Yu Z Wang B Early microbial and metabolomic signatures predict later onset of necrotizing enterocolitis in preterm infants. Microbiome. (2013) 1(1):13. 10.1186/2049-2618-1-13 24450576 PMC3971624

[22] Wang Y Hoenig JD Malin KJ Qamar S Petrof EO Sun J 16S rRNA gene-based analysis of fecal microbiota from preterm infants with and without necrotizing enterocolitis. ISME J. (2009) 3(8):944–54. 10.1038/ismej.2009.37 19369970 PMC2713796

[23] Normann E Fahlén A Engstrand L Lilja HE. Intestinal microbial profiles in extremely preterm infants with and without necrotizing enterocolitis. Acta Paediatr. (2013) 102(2):129–36. 10.1111/apa.12059 23082780

[24] Kayama H Okumura R Takeda K. Interaction between the Microbiota, epithelia, and immune cells in the intestine. Annu Rev Immunol. (2020) 38:23–48. 10.1146/annurev-immunol-070119-115104 32340570

[25] Lu P Sodhi CP Hackam DJ. Toll-like receptor regulation of intestinal development and inflammation in the pathogenesis of necrotizing enterocolitis. Pathophysiology. (2014) 21(1):81–93. 10.1016/j.pathophys.2013.11.007 24365655 PMC4067244

[26] Tompkins TA Hagen KE Wallace TD Fillion-Forté V. Safety evaluation of two bacterial strains used in Asian probiotic products. Can J Microbiol. (2008) 54(5):391–400. 10.1139/W08-022 18449224

[27] Wang X Yang Y Huycke MM. Risks associated with enterococci as probiotics. Food Res Int. (2020) 129:108788. 10.1016/j.foodres.2019.108788 32036912

[28] Stewart CJ Marrs EC Magorrian S Nelson A Lanyon C Perry JD The preterm gut microbiota: changes associated with necrotizing enterocolitis and infection. Acta Paediatr. (2012) 101(11):1121–7. 10.1111/j.1651-2227.2012.02801.x 22845166

[29] Huang S Rong X Liu M Liang Z Geng Y Wang X Intestinal mucosal immunity-mediated modulation of the gut microbiome by oral delivery of Enterococcus faecium against salmonella enteritidis pathogenesis in a laying hen model. Front Immunol. (2022) 13:853954. 10.3389/fimmu.2022.853954 35371085 PMC8967290

[30] Franz CM Huch M Abriouel H Holzapfel W Gálvez A. Enterococci as probiotics and their implications in food safety. Int J Food Microbiol. (2011) 151(2):125–40. 10.1016/j.ijfoodmicro.2011.08.014 21962867

[31] Sharif S Meader N Oddie SJ Rojas-Reyes MX McGuire W. Probiotics to prevent necrotising enterocolitis in very preterm or very low birth weight infants. Cochrane Database Syst Rev. (2020) 10(10):Cd005496. 10.1002/14651858.CD005496 33058137 PMC8094746

[32] Liu D Shao L Zhang Y Kang W. Safety and efficacy of Lactobacillus for preventing necrotizing enterocolitis in preterm infants. Int J. (2020) 76:79–87. 10.1016/j.ijsu.2020.02.031 32109650

[33] Robertson C Savva GM Clapuci R Jones J Maimouni H Brown E Incidence of necrotising enterocolitis before and after introducing routine prophylactic Lactobacillus and Bifidobacterium probiotics. Arch Dis Child Fetal Neonatal Ed. (2020) 105(4):380–6. 10.1136/archdischild-2019-317346 31666311 PMC7363787

[34] Mu Q Zhang H Liao X Lin K Liu H Edwards MR Control of lupus nephritis by changes of gut microbiota. Microbiome. (2017) 5(1):73. 10.1186/s40168-017-0300-8 28697806 PMC5505136

[35] Li YF Zhu CR Gong XL Li HL Xiong LK Wang KJ Beneficial effects of probiotic treatment on gut Microbiota in very low birth weight infants. Gastroenterol Res Pract. (2019) 2019:3682836. 10.1155/2019/3682836 31772570 PMC6854177

[36] Yang S Zhao Y Yu J Fan Z Gong ST Tang H Sugar alcohols of polyol pathway serve as alarmins to mediate local-systemic innate immune communication in drosophila. Cell Host Microbe. (2019) 26(2):240–51.e8. 10.1016/j.chom.2019.07.001 31350199

[37] Agius L Centelles J Cascante M. Multiple glucose 6-phosphate pools or channelling of flux in diverse pathways? Biochem Soc Trans. (2002) 30(2):38–43. 10.1042/bst0300038 12023820

[38] Gomis RR Favre C García-Rocha M Fernández-Novell JM Ferrer JC Guinovart JJ. Glucose 6-phosphate produced by gluconeogenesis and by glucokinase is equally effective in activating hepatic glycogen synthase. J Biol Chem. (2003) 278(11):9740–6. 10.1074/jbc.M212151200 12519761

[39] Ma J Wei K Liu J Tang K Zhang H Zhu L Glycogen metabolism regulates macrophage-mediated acute inflammatory responses. Nat Commun. (2020) 11(1):1769. 10.1038/s41467-020-15636-8 32286295 PMC7156451

